# Successful necrosectomy using the over-the-scope grasping device: a tool for solid necrotic debris

**DOI:** 10.1055/a-2443-4173

**Published:** 2024-11-08

**Authors:** Rahul Karna, Suchapa Arayakarnkul, Natalie Wilson, Mohammad Bilal

**Affiliations:** 1 Division of Gastroenterology and Hepatology, University of Minnesota Medical School, Minneapolis, United States; 2 Department of Internal Medicine, University of Minnesota Medical School, Minneapolis, United States; 3 Division of Gastroenterology and Hepatology, Minneapolis Veteran Affairs Medical Center, Minneapolis, United States

Direct endoscopic necrosectomy (DEN) can be a challenging procedure in patients with a large amount of necrotic debris. Traditionally, DEN has been performed with a combination of snares, baskets, forceps, and suction with intermittent irrigation. These instruments were not designed for necrosectomy and often slip off the necrotic tissue, making DEN cumbersome 1 . An over-the-scope (OTS) grasping device (OTSG Xcavator; Ovesco Endoscopy AG, Tübingen, Germany) has been designed for removal of necrotic tissue, facilitated by the extra-large grasper attached to the tip of the endoscope 2 . Here, we describe a case in which DEN was efficiently performed using the OTS grasping device.

A 63-year-old man with a history of semaglutide-induced necrotizing pancreatitis leading to an infected walled-off necrotic collection presented for DEN. The patient had undergone endoscopic transluminal drainage using 20-mm lumen-apposing metal stents (LAMSs) across cystgastrostomy for a 20-cm necrotic collection a week prior to presentation. Computed tomography scan showed the LAMS located across a large necrotic collection with solid debris ( Fig. 1 ). Esophagogastroduodenoscopy revealed a large amount of thick and pasty necrotic tissue adherent to the cyst wall, making DEN challenging with traditional tools including forceps, snares, and suction with intermittent irrigation. The OTS grasping device with grasping forceps was used to perform necrosectomy ( Video 1 ).

**Fig. 1:**
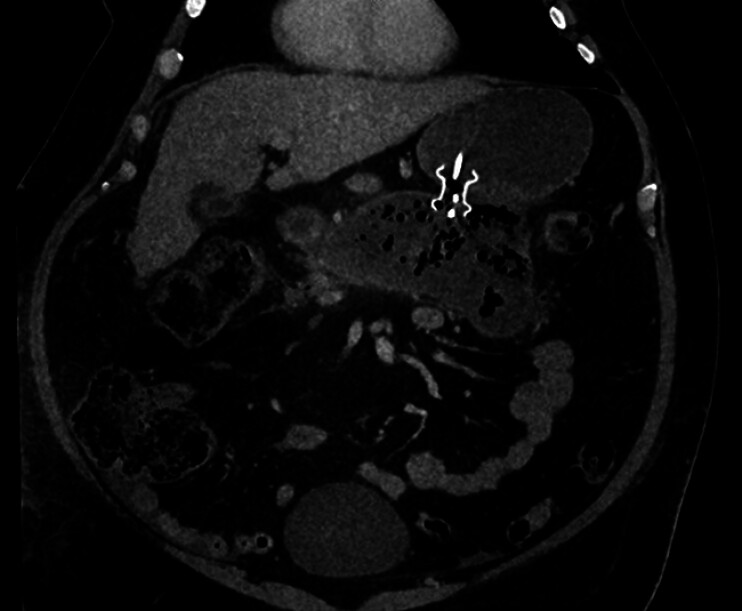
Computed tomography scan showing the lumen-apposing metal stent located across the cystgastrostomy and large walled-off necrotic collection.

Successful and efficient direct endoscopic necrosectomy using an over-the-scope grasping device in a patient with thick and adherent necrosum in a walled-off cavity.Video 1

Necrotic tissue was captured with grasping forceps and necrosum was pulled into the OTS grasping device. The device was closed and large pieces of necrosum, measuring 10–15 cm in length, were removed ( Fig. 2 ). Complete resolution of necrosis was achieved in two sessions ( Fig. 3 ). LAMSs were removed and replaced with plastic double-pigtail stents across the cystgastrostomy. The patient had no adverse events with the use of the OTS grasping device.

**Fig. 2:**
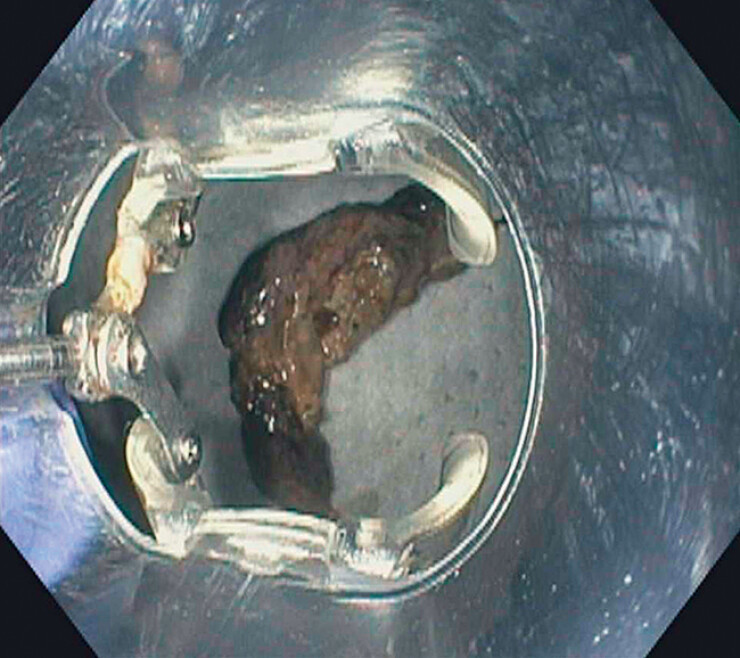
A large section of necrosum removed using the over-the-scope grasping device.

**Fig. 3:**
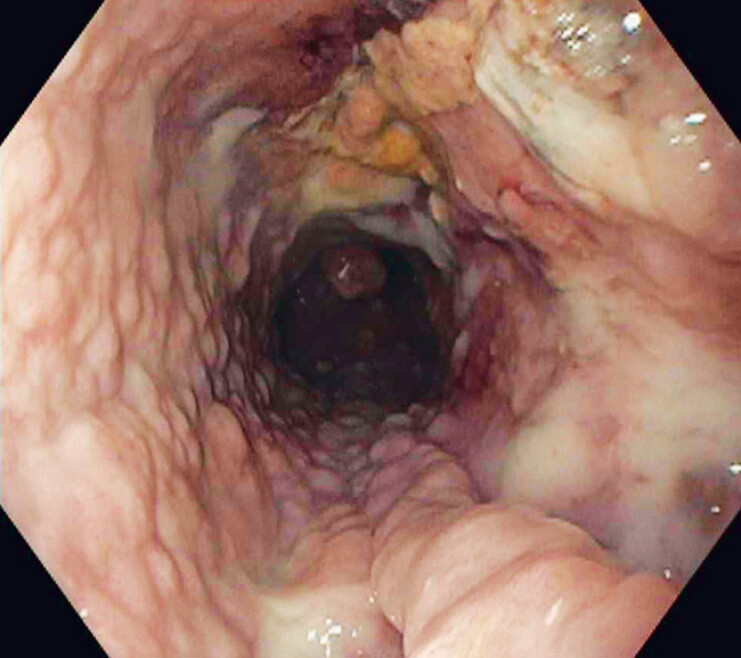
Complete debridement of the walled-off cavity was achieved using the over-the-scope grasping device.

Our case shows that the OTS grasping device is a safe and effective tool that can allow for efficient removal of large necrotic debris. Future studies are needed to assess the procedure efficiency of the OTS grasping device in comparison with conventional tools used in endoscopic necrosectomy.

Endoscopy_UCTN_Code_TTT_1AS_2AJ

## Notes

Endoscopy E-Videos https://eref.thieme.de/e-videos
*E-Videos* is an open access online section of the journal *Endoscopy* , reporting on interesting cases and new techniques in gastroenterological endoscopy. All papers include a high-quality video and are published with a Creative Commons CC-BY license. Endoscopy E-Videos qualify for HINARI discounts and waivers and eligibility is automatically checked during the submission process. We grant 100% waivers to articles whose corresponding authors are based in Group A countries and 50% waivers to those who are based in Group B countries as classified by Research4Life (see: https://www.research4life.org/access/eligibility/ ). This section has its own submission website at https://mc.manuscriptcentral.com/e-videos .
